# Social determination of alcohol consumption among Indigenous peoples in Colombia: a qualitative meta-synthesis

**DOI:** 10.1186/s12889-023-15233-6

**Published:** 2023-03-13

**Authors:** Canma Liliana Arévalo Velásquez, Jovana Alexandra Ocampo Cañas, María Teresa Buitrago Echeverri

**Affiliations:** 1 Health Systems, Childhood, Gender, Interculturality, and Tropical Diseases Research Track. Public Health, Medical Education, and Medical Professionalism Research Group. School of Medicine and School of Government, University of the Andes, Bogotá, Colombia; 2 Faculty of Health and Sports Sciences, University Foundation of the Andean Region, Bogotá, Colombia

**Keywords:** Alcohol consumption, Indigenous peoples, Colombia, Social Medicine, Meta-synthesis

## Abstract

**Background:**

Alcohol consumption is a social phenomenon that involves society, groups, and individuals from different cultures around the world. Among some Indigenous groups located in Colombia, South America, alcohol consumption has been present in their lives, where contradictory processes occur and generate public health attention. We aimed to analyze qualitative research findings on alcohol consumption among Indigenous peoples in Colombia.

**Methods:**

This article used the qualitative meta-synthesis methodology, which included: (a) comprehensive search strategy, (b) appraisal of qualitative research reports, (c) findings classification, and (d) synthesis. Databases were searched for papers published from 2004 to 2019 in SCOPUS, LILACS, PROQUEST, and JSTOR, among other sources of information. A total of 2,159 papers were reviewed and finally, 13 studies were included in this meta-synthesis. The synthesis of findings included a constant comparative analysis and also aimed for the articulation of its findings to alternative perspectives in a predefined matrix.

**Results:**

Nine Indigenous ethnic groups of Colombia were represented in the 13 articles analyzed. From the analysis emerged the symbolic approach “Alcohol: a chameleon that unpredictable society colors” as the meta-theme of this research. This reflects four social processes that influence interaction with alcohol: Dynamic Systems Mergers (Indigenous system, influence of non-Indigenous system); Diverse Authority Spheres (parenting, Indigenous authority, school, university, religious and spiritual, traditional medicine); Between Transculturation and Interculturality (cultural crises effects and dynamism); and the Paradoxes of the Normalization of Alcohol (reasons, functions, and types of alcohol consumption). Likewise, these results support the social determination of health and sociocultural epidemiology perspectives, as being an adequate way of explaining a complex phenomenon.

**Conclusion:**

Alcohol consumption among Indigenous peoples in Colombia is a social construction. Alcohol acts as an instrument, which is present in the changing relationships and tensions of social processes. This is reflected in harmonies, or disharmonies, in the life of Indigenous Colombians, which take place in a historical, sociocultural, economic, and political context. The results provide a reference point to guide practice and research but also reiterate the need to include the social determination of health perspective in public policies, as a path to the understanding of alcohol consumption.

**Supplementary Information:**

The online version contains supplementary material available at 10.1186/s12889-023-15233-6.

## Background

Diverse cultures around the world have considered alcohol consumption a normal part of their lives. Thought, the excess of this substance impacts public health and social development. For example, among Indigenous peoples in the Americas, the prevalence of problematic alcohol consumption has been reported between 86.5% and 95% [1, 2]. Also, high alcohol consumption has been involved in social problems (aggressiveness, problems at work, car accidents), organic problems (death, sweating, liver problems, weakness), and specifically deaths due to liver cirrhosis at rates of 82.2%, 80%, and 5.5%, respectively [3]. Furthermore, there is a greater burden of disease in low- and lower-middle-income countries [4], which generates social and health differences.

In Colombia, Indigenous peoples constitute 4.4% of the population [5]. A total of 115 ethnic groups are recognized. They are distributed around 79% in rural areas and 21% in urban areas throughout the departments of Colombia [5]. The greatest concentration is located in La Guajira, Cauca, and Nariño. Wayuu, Zenú, Nasa, and Pastos ethnic groups represent 58.1% of the Indigenous peoples [5]. Regarding morbimortality related to alcohol consumption, a complete picture is not yet available. According to Colombia’s National Public Health Surveillance System, between 2009 and 2014, 118 cases were reported of Indigenous peoples who were at risk, or with mental and behavioral disorders, due to the use of alcohol and other psychoactive substances. Additionally, deaths from chronic liver diseases and cirrhosis were reported [6] that could be related to this consumption.

The magnitude of alcohol damage is influenced by poverty [7]. Poverty rates for Indigenous Colombians are higher compared to other population groups (26.6%) [8]. It has been shown that in countries with higher income and economic development, alcohol consumption rates are greater. However, it is the poorest countries, that tend to suffer greater mortality, disability [7], and social burden, even if they consume the same amount of alcohol.

A study of Colombian Indigenous peoples reported that alcohol was the substance with the highest annual consumption (81.6%) in people aged 18 years and older [8] and was higher than the national rate (42.5%) [9]. Indigenous peoples have presented alcohol problem prevalence at a rate of 16.2%, distributed as excessive (8%), risky (7.9%), and probable dependence (0.3%) [8]. Ethnic studies have reported the prevalence of problem drinking mostly in men with a 50% and 72% in urban and rural, respectively [10, 11].

Despite these advances, there are still missing pieces in the research literature. This review found that, although there are qualitative studies with findings on alcohol consumption among Indigenous peoples in Colombia, no meta-synthesis has been carried out with them. In other words, the findings of the qualitative studies had not been analyzed nor had an interpretative synthesis been generated with them.

The closest studies using this meta-synthesis method have focused on the recovery of alcohol addiction in non-Indigenous peoples [12], or on violence, which included a study with Colombian Indigenous peoples [13]. We consider it important to conduct a meta-synthesis because the findings on alcohol consumption among this population must be understood more broadly and deeply given the complexity of the phenomenon.

In line with the above, it leads us to consider going further with the results of the meta-synthesis. These results are an opportunity for a deductive analysis with the social determination of health perspective [14] and its complementarity with sociocultural epidemiology [15]. These perspectives rethink the results of alcohol consumption among Indigenous peoples in Colombia from the complexity of the health-disease process.

A classical epidemiological perspective is often observed, which is limited to the causality given by risk and protective factors [14]. The social determination of health (a category of critical epidemiology) does not preclude the initial approach to risk factors [16]. However, just making this approach to health problems leads to the practice being reduced to limited functional actions on risk factors [14].

In particular, the analysis of alcohol consumption among the Indigenous peoples of Colombia, based on the social determination of health, makes visible several aspects that occur in an adverse historical, social, cultural, economic, and political context.

For example, it helps to explain the hierarchical relationships that are generated between a capital accumulation system with the ways of life of groups and individuals. It also explains the differential vulnerability that may be present in the power relations that exist in terms of gender, ethnicity, and social class. These aspects become crucial in the epidemiological distribution [17, 18] in this case, in drinking or not drinking alcohol among Indigenous peoples of Colombia.

This perspective is complemented by sociocultural epidemiology [15]. This captures relevant aspects of reality such as the alcoholization process [15, 19], the multifunctionality of alcohol, and the negative consequences [20, 21] for society, Indigenous groups, and individuals. These groups are characterized by their heterogeneity and cosmovisions (ways of seeing and conceiving the world), in which alcohol consumption is part of the relationships that operate in the processes of domination and subordination [22]. Thus, alcohol consumption is understood as “the intake, within a process that institutes it, and gives it specific functionalities, within the play of dominant relations in each society or culture” [23].

In summary, it is important to carry out a study in which the results of the meta-synthesis and the proposed perspectives are integrated to broaden the understanding of alcohol consumption among Indigenous peoples in Colombia. Being open to diverse approximations and perspectives that explain the results of this meta-synthesis is part of the path toward the transformation of this complex phenomenon.

The main aim of this meta-synthesis [24] is to analyze the qualitative research findings on alcohol consumption among Indigenous peoples in Colombia during the last fifteen years. To achieve it, these are the particular objectives:

- To review systematically qualitative research on alcohol consumption among Indigenous peoples in Colombia between 2004 and 2019.
- To conduct the interpretative integration of the findings of qualitative research on alcohol consumption among Indigenous peoples in Colombia between 2004 and 2019.
- To articulate the results of the meta-synthesis with the theoretical categories predefined in the matrix called Epidemiological and sociocultural profile of alcohol consumption among Indigenous peoples in Colombia.

Two principal questions guide this research. First: How have qualitative research findings reported alcohol consumption among Indigenous peoples in Colombia between 2004 and 2019? Secondly: Are the social determination of health and its complementarity with sociocultural epidemiology an adequate way to explain the identified social processes?

## Methods

A meta-synthesis of qualitative studies was conducted. This involved the interpretative integration of the findings, which are themselves interpretative syntheses of data [24]. This made it possible to create larger and more faithful representations of the richness of the primary findings [25].

The development of the study followed the ENTREQ [26] guidelines and the procedural phases of Sandelowski and Barroso [24]. Other authors also complemented it. These included: (a) comprehensive search strategy, (b) appraisal of qualitative research reports (c) findings classification, and (d) synthesis. The information on the phases was registered and organized using the matrix method [27].

### Comprehensive search strategy

The literature search process was carried out between September and October 2020. It was based on the elements proposed by the STARLITE mnemonic [28] (see Table 1).

**Table 1:** STARLITE: elements applied in systematic literature searches

**S: Sampling strategy**	Comprehensive on the phenomenon of interest and type of studies
**T: Type of studies**	Fully reported (primary qualitative studies and mixed-methods studies in which qualitative findings could be separated from quantitative findings)
**A: Approaches**	Approaches other than electronic subject search: manual searching using Berrypicking techniques [29]
**R: Range of years**	Fully reported from 01- 2004 until 12-2019. (this range is part of the inclusion criteria)
**L: Limits**	Language (Spanish and English)
**I: Inclusion and exclusions**	**Inclusion**: (a) qualitative methods with findings of the phenomenon under study, (b) other related phenomena with findings of the phenomenon under study, (c) published or unpublished studies, with or without a peer review, gray literature, (d) multidisciplinary studies. **Initial exclusion**: (a) quantitative method, (b) reports with no data related to alcohol drinking, (c) thematic reviews, (d) secondary reviews. **Second exclusion**: (a) no ethical aspects (b) no findings on alcohol drinking (c) similar results in the same ethnic group (d) language limitations.
**T: Terms used**	Example of a sample search strategy from PROQUEST: S1: “indigenous people$” OR “indigenous” OR “Aboriginal$” OR “native$” OR “ethnic$”. S2: “Alcohol drinking” OR “alcoholic beverages” OR “Alcohol use” OR “alcohol$” OR “chicha” OR “liquor”. S3: “Colombia”. S1 AND S2 AND S3: (“indigenous people$” OR “indigenous” OR “Aboriginal$” OR “native$” OR “ethnic$”) AND (“Alcohol drinking” OR “alcoholic beverages” OR “Alcohol use” OR “alcohol$” OR “chicha” OR “liquor”) AND “Colombia”
**E: Electronic sources**	**(1) Databases**: SCOPUS, LILACS, PROQUEST, JSTOR. **(2) Web search engine** Google Scholar (first 10 sheets). **(3) Institutional Repositories**: FLACSOAndes, CINDE, URACCAN, Uniandes, UNAL, UDEA, UNIVALLE, UNICAUCA. **(4) Specialized Journals online**: Revista Colombiana de Psiquiatría, Revista Facultad Nacional de Salud Pública, Revista de la Facultad de Medicina UNAL, Revista de Estudios Sociales, Revista Antídopa, Revista Ciencia e Interculturalidad. Of the 34 pre-reviewed electronic resources,19 were selected.

The exhaustive sampling strategy broadened the sensitivity of the searches. This required manual searches [29] and electronic sources, with different search strategies and adaptations of the words of the primary research question. In databases with the thesaurus, controlled terms were identified (DeCS, EMTREE, MeSH), while in other databases and complementary sources, free terms were used. Boolean (AND, OR) and truncation (*$) operators were combined (see Additional file 1). All papers were screened by one reviewer (CLAV) and validated by an expert librarian whereby differences were resolved by discussion. The retrieved records were exported to Mendeley to manage citations and remove duplicates.

**Fig. 1:**
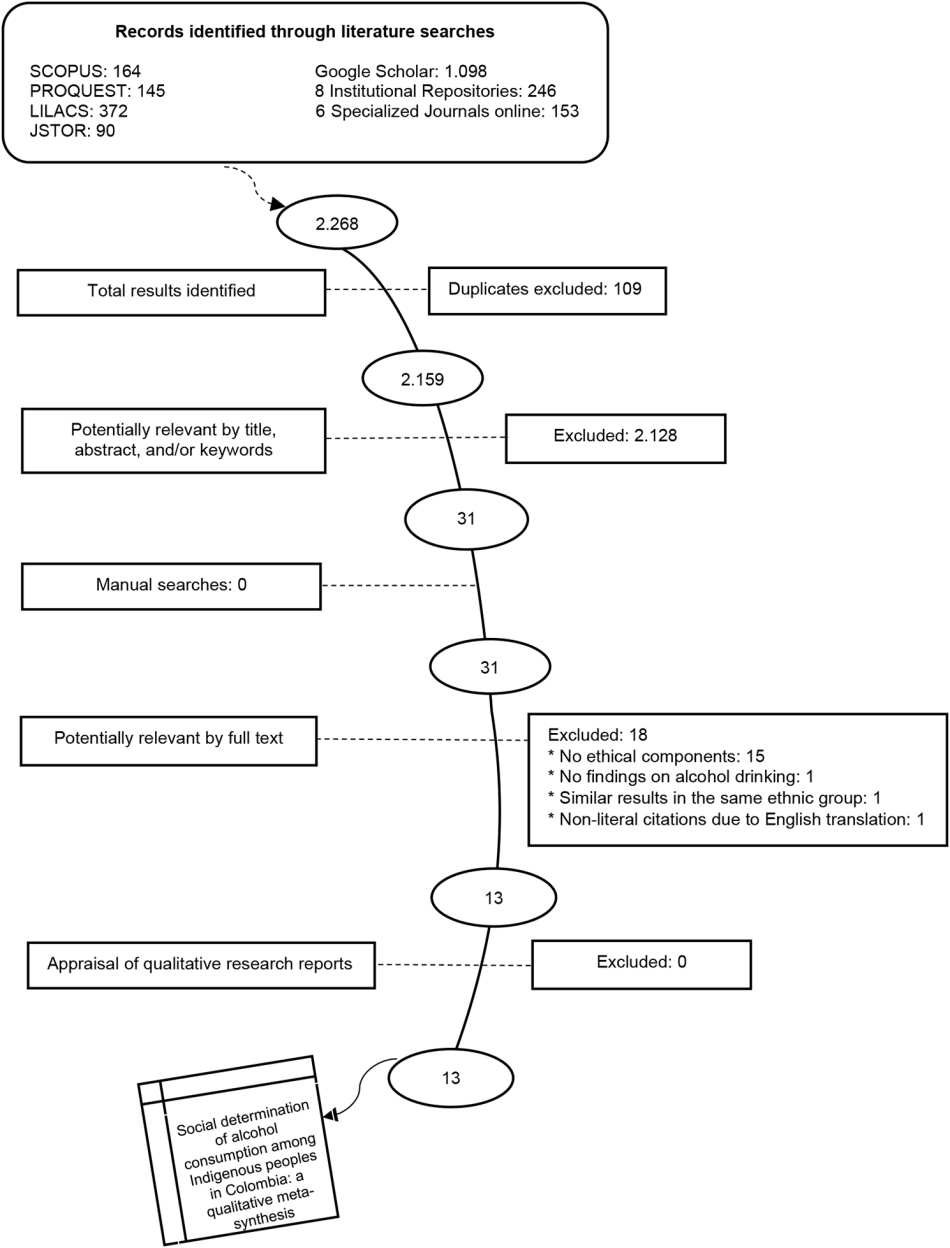
Results of the literature search

This resulted in 2,159 studies which were screened by title, abstract, and/or keywords from the entire text. (Fig. 1). All studies were assessed based on the eligibility criteria. The inclusion criteria were:

- Studies published from January 2004 to December 2019: this time range was due to various reasons: (a) the initial reference was the official data from 2009 to 2014 with a quantitative analysis, which included within the public health events the consumption of psychoactive substances such as alcohol in Indigenous peoples of Colombia [6], (b) however, there is an underreporting of the health situation of this population before 2009 [6]. Based on this, it was decided to go back five years (i.e., since 2004) in the search for qualitative studies. In this search, we found one qualitative study between 2004 and 2009 that met the eligibility criteria. This search result, added to the time required to search further back in time, led to defining 2004 as the starting date. Meanwhile, the year 2019 as the closing date allowed us to understand the phenomenon under study five years after the official quantitative data [6] and was the full year immediately before the beginning of the research.
- Primary qualitative studies (grounded theory, case studies, ethnography, phenomenology, descriptive study, participatory action research, content analyses, etc.) with findings on alcohol consumption among Indigenous peoples from specific ethnic groups in Colombia.
- Mixed-methods studies (when qualitative findings could be separated from quantitative findings).
- Other related phenomena with findings related to alcohol consumption among Indigenous peoples.
- Published or unpublished studies, with or without a peer review, gray literature (theses and dissertations).
- Studies made by diverse disciplines affiliations (public health, social medicine, community health, nursing, psychology, sociology, anthropology, education, politics, and others.)

The initial exclusion criteria were:

- Quantitative method, reports with no data related to alcohol consumption among Indigenous peoples from specific ethnic groups in Colombia, thematic reviews, and secondary reviews.

The second exclusion criteria were:

- no ethical aspects (no signs of informing participants of the purpose of the study, prior consent/consultation, risks and benefits of participation approach traditionally or formally).
- no findings on alcohol drinking (there was not even a single direct quote-finding set in the entire document).
- similar results in the same ethnic group (the identification of a greater variety of alcohol-related themes in ethnic groups is prioritized, i.e., reflecting as many of the themes).
- language limitations (no literal citations due to their translation into another language).

This search strategy identified 13 studies [30–42]. (Fig. 1). To find them, it was necessary to read and exclude a considerable number of documents that were not very relevant, in the search for studies with characteristics relevant to the research. This allowed us to have a representativeness of the phenomenon to be interpreted [43] and an in-depth analysis [24, 44].

### Appraisal of qualitative research reports

The 13 studies had individual and comparative appraisals [24]. The individual appraisal permitted familiarization with the content of the study, visualization of its location, and a reflective exercise on the value of each study [24]. This appraisal included a critical reading [24] and a review of trustworthiness criteria [45]. The critical reading included aspects such as research purpose and question(s), sampling, data collection and analysis, techniques for maximizing validity, findings, discussion, and implications [24]. Likewise, a Critical Appraisal Form Guidelines for qualitative studies, based mainly on descriptions by Lincoln and Guba was used (see Additional file 2). In this critical reflection, four trustworthiness criteria were reviewed: credibility, transferability, dependability, and confirmability [45], and complemented with the criteria of ethics and the general relevance of each research.

These criteria were appraised independently by two researchers (CLAV and MTBE), and all researchers (CLAV, MTBE, JAOC) discussed the results. No research was excluded based on the quality of their reporting. According to Sandelowski and Barroso [46], no consensus exists concerning quality neither in qualitative research nor on the use of quality criteria in systematic reviews.

Furthermore, Sandelowski argued that “the process of judging inevitably entails the active deployment of taste or the selection of those considerations deemed applicable to anyone object of evaluation in addition to the continual (re) interpretation of those considerations and their applicability to any other comparable object of evaluation”[47]. The 13 studies were left by consensus based on the aim of the research and the value of the findings for this meta-synthesis. (Fig. 1). It is worth mentioning that this individual appraisal process is articulated with the finding’s classification phase [24].

After the individual appraisal of each study was completed, the comparative appraisal across the reports was done. The comparative appraisal allowed us to visualize the data as a whole and to recognize patterns or trends [24]. The relevant characteristics of the studies were registered in a Microsoft Excel spreadsheet and discussed the results (CLAV, JAOC). (see Table 2).

**Table 2:** Characteristics of the studies included

Study #	First Author, Year	Ethnic group	Colombian geographic location	Participants (*)	Specific inclusion criteria	Study Design	Disciplinary affiliation
1	Panqueba (2005) [30]	Muisca	Urban and rural areas in Bogotá	women and men, young, adults and elderly	Everyday memory and historical identification with alcohol consumption findings.	Descriptive ethnographic	Social Sciences
2	Yepes et al. (2010) [31]	Embera	Indigenous reservation in Antioquia (rural community)	women and men, young, adults and elderly	Gender and intergenerational violence with findings on alcohol consumption.	Participatory Action Research (PAR)	Epidemiology, Education, Psychology, Dentistry, Public health
3	Oca et al. (2014) [32]	Nasa	Indigenous reservation in Cauca (rural community)	women and men, children, young, adults, and elderly	Social impacts of excessive drinking of chicha de caña (sugar cane beverage)	Unspecified (follows PAR principles)	Education
4	Ruiz (2015) [33]	Embera Chamí y Katío	Urban area in Bogotá	* Chamí: (4 women), (2 women and 2 young adult men). 28 were surveyed (11 women and 17 young and adult men). * Katío: (2 women and 2 young adult men). 37 were surveyed (20 men and 17 women, young, children). Also 4 Indigenous and 4 non-Indigenous experts (1 national and 1 international).	Relationship between armed conflict and mental health in situations of forced displacement with findings on alcohol consumption	Mixed qualitative techniques	Public health
5	González (2016) [34]	Wayuu	Rural and urban areas in the Guajira	4 women and 4 men; aged ≥ 18 years	Central Human Capabilities with findings on alcohol consumption	Exploratory and interpretive qualitative	Education
6	Campo (2017) [35]	Nasa	Indigenous reservation in Cauca (rural community)	young women and men (around 9)	Cultural practices that influence alcohol consumption	Biographical	Education
7	Lozano (2017) [36]	Embera Chamí	Indigenous reservations in Caldas (rural community)	481 among young, adult, and older women and men. Between 24 to 26 women per Indigenous reservation.	Social determination of health in childhood with findings on alcohol consumption.	Mixed approach with qualitative orientation	Human and social sciences
8	Bohórquez et al. (2017) [37]	Ticuna	Indigenous reservation in Amazonas (rural community)	1 woman and 2 men, about 50 children, and adolescents	Psychosocial risk and protective factors for suicidal behavior with findings on alcohol consumption	Discourse analysis	Psychology
9	Leal et al. (2017) [38]	Pijao	Indigenous reservations in Tolima (rural community)	5 men	Managing Psychotic symptoms by ancestral doctors with findings on alcohol consumption	Qualitative interpretative	Psychiatry
10	Posada et al. (2017) [39]	Zenú and others	Urban area in Antioquia	16 young men and women participants, only 4 of them Zenú	Psychoactive substances and university prevention programs with findings on alcohol consumption	Grounded theory	Public health
11	Camacho et al. (2017) [40]	Kamëntšá	Indigenous reservation in Putumayo (rural community)	8 boys and girls, 12 women and men, and elderly	Community representations of childhood with findings on alcohol consumption	Phenomenological based Ethnographic	Education, social sciences
12	Cipamocha et al. (2018) [41]	Ticuna	Indigenous reservation in Amazonas (rural community)	about 40 women and men, including children, young and adults	Social support networks to decrease suicidal behavior in children and youth with findings on alcohol consumption	Content analysis	Psychology
13	Zacipa et al. (2019) [42]	Misak	Indigenous reservation in Cauca (rural community)	13 women, 10 men, and other women (girls, adolescents, adults, elderly)	Pentecostal women with findings on alcohol consumption	Ethnography	Human and social sciences

(*) Approximately 756 participants. Not all studies detailed this data

### Findings classification

Before classifying the findings, the 13 studies were read and reread. Most of the findings were placed in the results or discussion sections. The findings were then extracted from the primary authors in a matrix, preceded by direct quotations from the participants, and coded before identifying the typology of the findings. An example can be found in Additional file 3.

Classification by type of finding determined the degree of transformation of the raw data by the researcher [24, 48]. Of the 200 preselected data, 177 were identified with a degree of transformation and considered as findings. The preselected data were classified: (a) 41 there was an effort to go on to describe the themes (thematic survey), (b) 136 provided findings in the form of conceptual/thematic description or advanced to interpretive explanations, and (c) 23 data with no transformation, i.e., no findings. It was performed independently by two researchers (CLAV and MTBE), and all researchers (CLAV, MTBE, JAOC) discussed the results and reached a consensus to leave the 13 studies. This typology of findings was a key component during the appraisal critical process, which favored inclusion based on the relevant content of the phenomenon and its importance in orienting the practice [24, 48].

### Synthesis process

This process combined an inductive analysis with the meta-synthesis and a deductive analysis based on the categories predefined for the theoretical referents (Breilh [17, 18, 49], Menéndez and Cortés [15, 19–21].

**Fig. 2:**
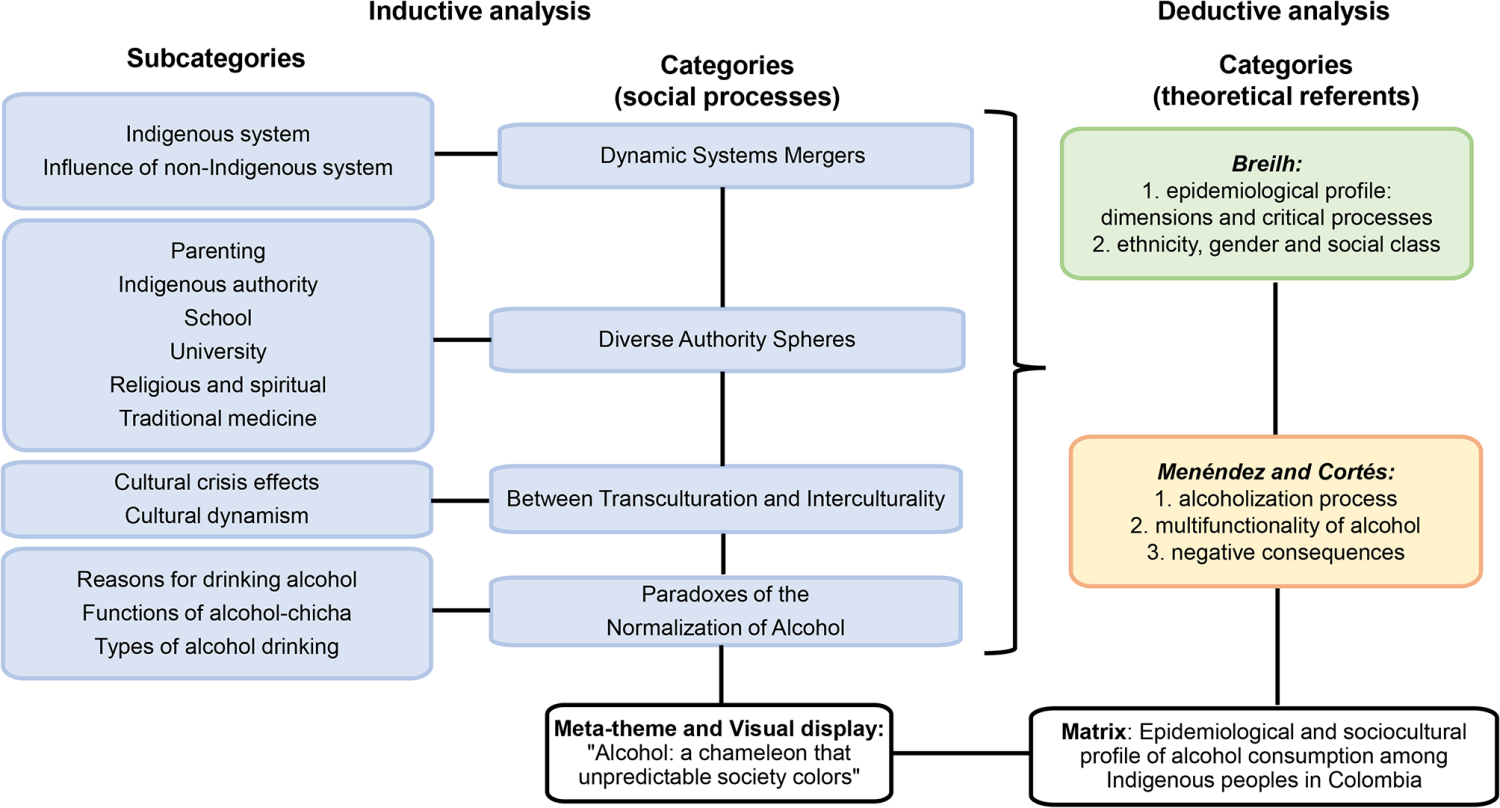
Results of the findings synthesis process (Source: Created by the authors)

First, the inductive analysis consisted in reviewing line-by-line 177 findings, and with open coding, 45 codes were obtained. This was done through constant comparative analysis in search of similarities and differences [50], the use of imported and in vivo concepts, metaphors[24], and memos by two reviewers (CLAV, MTBE). With axial coding [50], 13 subcategories were obtained, which were integrated into four categories (social processes). These are related to each other and provide a more precise and complete explanation of the phenomenon [50] (Fig. 2). The analysis was continuously discussed among the researchers (CLAV, MTBE, JAOC).

The previous coding involved systematic relationships between categories [50, 51], which formed the basis for the development of interpretations [51]. This meta-synthesis expanded in a novel way the interpretative possibilities of the findings [24] and integrated them as a whole [24, 51]. This led to researcher CLAV going further [51] and generating a central category [50] (named meta-theme) [52] (see Fig. 2), that answers the primary research question.

The meta-theme “Alcohol: a chameleon that unpredictable society colors” brought the categories together to form an explanation as a whole [50]. Its definition entailed the use of metaphorical language richness [50] and the connection with nature that Indigenous cultures have. It is a way, through which they try to explain their relationships with everything that surrounds them and with themselves.

**Fig. 3:**
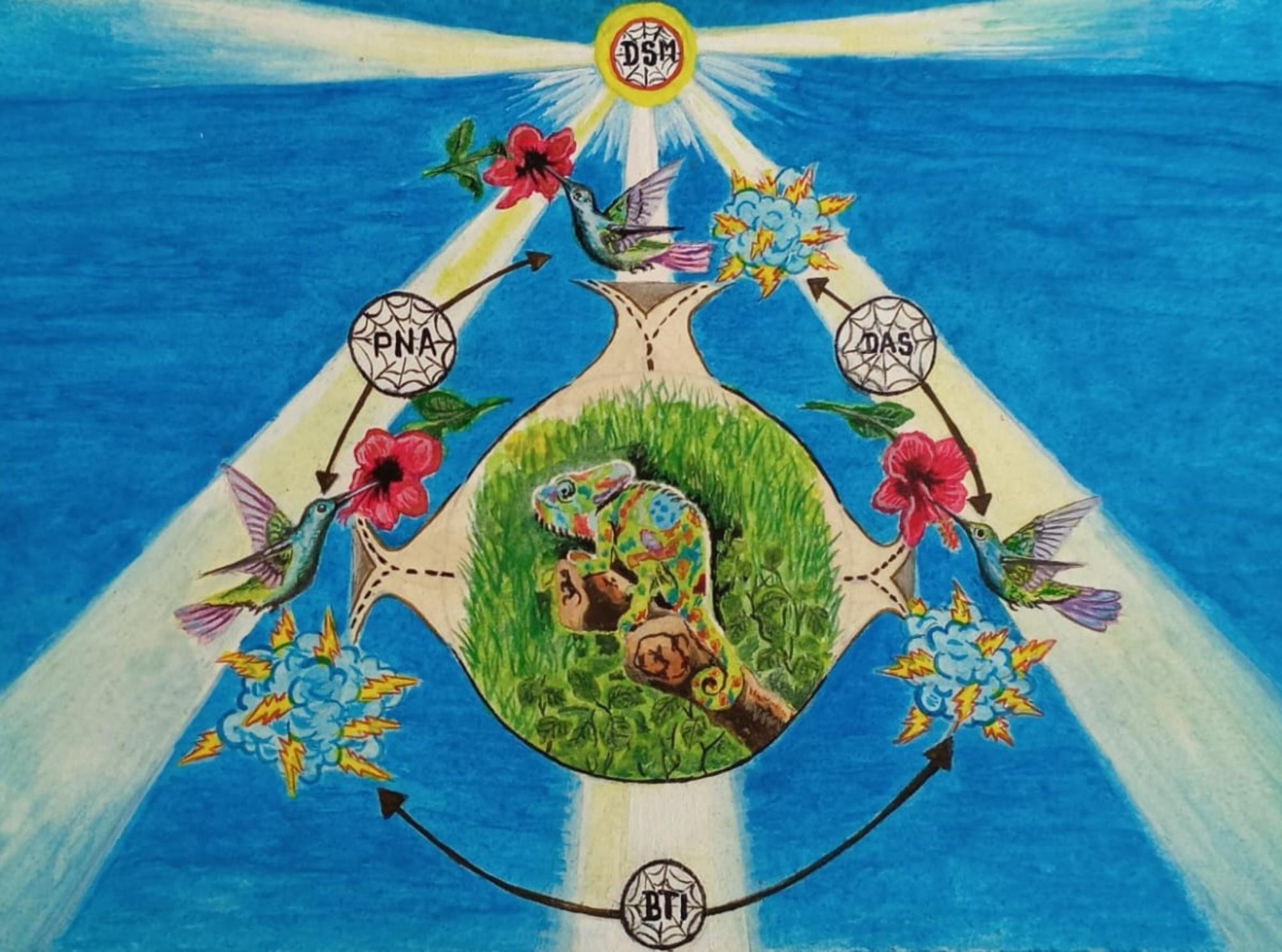
Visual display of the meta-theme: “Alcohol: a chameleon that unpredictable society colors”. (Source: own visual display. Illustrated by Indigenous scientist Confucio Hernandez Makuritofe)

The identification of the meta-theme and integration of the concepts was done using the techniques of writing a story argument and the diagram [50, 51] (named visual display) [24] (Fig. 3). The technique of writing a story argument allowed to see how the social processes (categories) vary and relate to each other. Thus generating a new interpretation [50] of alcohol consumption among the Indigenous peoples in Colombia.

Likewise, a visual display [24] of the meta-theme was created (Fig. 3). It made it possible to visualize the logic of the relationships [24, 50], the depth, and the complexity of thought [50]. Having this device in this meta-synthesis allows readers to focus on the key dimensions (social processes) of the phenomenon under study [24]. A visual display tends to generate closeness between the research participants and the synthesis researchers [24]. The validity of the integration produced is also supported, thus becoming a powerful rhetorical device [24]. It was created by researcher CLAV.

This metaphorical synthesis (Fig. 3) expanded the understanding of alcohol consumption among the Indigenous peoples in Colombia. The identification of social processes and a meta-theme condenses central aspects that influence the interaction with alcohol. This approach generated significant data that go beyond the description or summary of primary studies [26]. Instead, it involved leaps of imagination in an attempt to communicate the ideas as well as possible [24].

Second, the deductive process consisted of pouring the social processes identified in the meta-synthesis into a matrix. This matrix was constructed to articulate the social processes with predefined categories according to the theoretical referents (Breilh [17, 18, 49] Menéndez and Cortés [15, 19–21] (Fig. 2) (Table 3). Making this articulation visible is important because it allows us to understand how the social processes that influence the interaction with alcohol (identified in the meta-synthesis) are part of a structural context, ways of life, and lifestyle of the Indigenous peoples in which meaning is given to drinking or not drinking alcohol. (see Fig. 2; Table 3). The analysis was discussed among the researchers (CLAV, JAOC).

### The theoretical framework of the deductive analysis

The predefined categories (Fig. 2) have a meaning within the matrix. First, Breilh with the social determination of health recovers the complexity and interrelation of the health-disease process in a specific historical context [14]. This explanation of health as a dynamic and multidimensional social process can be understood by using the epidemiologic profile [14]. The profile consolidates three dimensions of the social determination of health: the general (society as a whole), the particular (groups), and the singular (individuals), in which the critical protective and destructive health processes are found [49]. The differences between these health processes are determined by gender, ethnicity, and social class[17, 18] whose power relations are important in the epidemiological distribution, in this case by drinking alcohol or not. (see Table 3)

Second, Menéndez with sociocultural epidemiology [15] complements the previous perspective. It was possible to identify in Menéndez’s [15, 19, 20, 22] and Cortes’ [19, 21] contributions a multidimensional thought of reality. From the general dimension, the economic-political and sociocultural processes establish the dominant characteristics of alcohol drinking or non-drinking, which is named the alcoholization process [15, 19] In the dimension of social groups, it is possible to observe cultural practices and the multifunctionality of alcohol [20, 21]. In the individual dimension, there are negative consequences [20, 21], which are also present in the dimensions of social groups and the alcoholization process; however, in these two dimensions, there are cultural processes that are protective (see Table 3).

The result of this integration perspective was named Epidemiological and socio-cultural profile of alcohol consumption among Indigenous peoples in Colombia (see Table 3). It is important to read the reality of alcohol drinking among Indigenous peoples from the dimensions of life. This influences the health and disease processes of groups and individuals in contradictory ways. Visualizing in a matrix the socio-cultural processes of alcohol consumption from the structural, group and individual dimensions broadens the understanding and answers the second research question. The analysis was discussed among the researchers (CLAV, JAOC).

## Results

This section details two main results. First, the synthesis of the 13 articles, which includes the interpretative integration of the findings present in the four social processes (categories) (Fig. 2), the meta-theme, and the visual display (Fig. 3). Second, the articulation of the social processes identified in the meta-synthesis with the categories predefined for the theoretical referents. This is shown in a matrix named Epidemiological and socio-cultural profile of alcohol consumption among Indigenous peoples in Colombia (Table 3).

### Study characteristics

In total there were 13 studies with findings of alcohol consumption among Indigenous peoples in Colombia [30–42]. Their characteristics are presented in Table 2. About 756 men and women participated, including children, youth, adults, and the elderly; exact data were not detailed in all studies. Nine Indigenous peoples were identified Muisca [30], Embera Chamí and Katío [31, 33, 36], Nasa [32, 35], Wayuu [34], Pijao [38], Zenú [39], Kamëntšá [40], Ticuna [37, 41], and Misak [42]. These ethnic groups are located in 8 different departments of Colombia. Nine in rural, two in urban, and two in urban and rural areas.

It was possible to identify that some studies [30, 32] were conducted by Indigenous researchers. The majority of the studies were from a qualitative perspective, with 12 qualitative studies, and one mixed method. Two studies focused on alcohol consumption [32, 35] and 11 studies focused on diverse phenomena related to the research topic. The phenomena were: memory and historical identification [30]; gender and intergenerational violence [31]; armed conflict and mental health [33]; central human capabilities [34]; social determination of health in childhood [36]; suicidal behavior [37, 41]; psychotic symptoms[38]; psychoactive substances and university prevention programs[39]; community representations of childhood [40]; and Pentecostal women [42]. Furthermore, eight different disciplinary affiliations were found (see Table 2).

### Description of social processes

We identified four social processes in the lives of Indigenous peoples in Colombia, that influence interaction with alcohol (1) Dynamic Systems Mergers; (2) Diverse Authority Spheres; (3) Between Transculturation and Interculturality; and (4) Paradoxes of the Normalization of Alcohol. Each of the four social processes is presented in more detail below.

#### Dynamic Systems Mergers

The essence of the Indigenous system has been based on its cosmogonies (mythical narratives relating to the origins of the world) and cosmovisions (ways of seeing and conceiving the world) [35]. This system has a history of self-sufficiency in medicine, architecture, manufacturing, and its mingas (cooperative and voluntary work) [32]. There have been advances in the legalization of Indigenous reservations, their government [36], and education [32], and they strive to maintain their practices and customs [36]. Nevertheless, there is housing in rural areas in precarious conditions, and poor nutritional situations [35].

In general, the Indigenous system has been influenced by a non-Indigenous system. The armed conflict [33] and Western culture have led to the loss of their cultures [32, 33, 35, 36] and their territories [31, 33]. Poor health care [31] and the lack of economic resources [31, 32] have caused young people to drop out of school and look for jobs [32, 35] such as in gold mining [32]. Likewise, the predominance of the working class continues in rural and urban areas [30–42].

The tensions between these systems, at the collective and individual levels, have influenced the relationship that they have with alcohol[35, 36]. This dates back to the history of the prohibition of chicha (fermented (alcoholic) or non-fermented beverage made from corn, and sugar cane, among others) by the Western system [35]; as well as, to the current national laws of education and childhood and adolescence, which the Indigenous consider disruptive in the parenting of their children [35–37, 40]. Another tension is related to the globalization[35], which has generated a culture of consumerism and, especially in Indigenous populations, the drinking of industrialized alcoholic beverages[35], which is preferred for young people in these communities [35, 36].

Healthy activities such as sports or recreational events are limited. In rural areas, their implementation has been conditioned by the purchase of alcoholic beverages, which sponsors and rewards these events organized by the authorities [31, 33, 35]. A participant reflects as follows: “The health service is destined to treat illnesses. There is no prevention system; on the contrary, the State promotes drinking and distribution of alcoholic beverages to pay for health and education services” [35].

In urban areas, those displaced by the conflict also have a lack of healthy options, lack of job opportunities, and live in conditions of poverty, which have left children and youth vulnerable to environments with the presence of drugs, alcohol, and acts of violence [33].

#### Diverse Authority Spheres

The authorities that are present in parenting, Indigenous government, school, university, religion, spirituality, and traditional medicine, move in a protective, but, also, conflicting, and contradictory manner around drinking, or not drinking, alcohol. In parenting, the prevention of excessive drinking has been sought based on the specific knowledge of each culture [32, 35], the advice of older adults, which is taken from ancestral care [40], and health education [36]. Although there are parents who say they do not have the tools to prevent [31], and even feel that they have lost their way in their family roles [35]. There is also the fear of those who raise their children in the city, who ask questions such as: “Dad, why do you talk to me about so many different things when they teach me other things at school? " [33]. Some parents feel that the state regulations go from protection to overprotection [35–37, 40]. Moreover, the presence of various situations in parenting, such as old-fashioned demands [35] permissiveness in drinking alcohol [35–37, 40] or the neglect of children, especially by parents who drink alcohol [32, 35, 37] or those who do not participate in parenting [31].

The Indigenous authorities are concerned about excessive drinking and the damage it causes to the community. They have created spaces for training [32, 35, 36] and regulation of the sale of alcohol to minors [35]. There is an application of traditional norms [32] but also permissiveness in community events that promote drinking, with leaders who do not set an example [31, 32, 35]. Some members of the community feel little attention, support, and discussion [31, 32] of this problem. As well as a lack of follow-up and contradiction in compliance with gender and generational equity norms [31].

At school and university, there are different ways of exercising authority. At school, some consider that the authoritarian education of the past was better[35], while the permissive education is associated with the current state norms because they lack discipline with the children [31, 32, 35]. Meanwhile, Indigenous education has left encouraging results related to excessive drinking [32]. At the university, drinking alcohol among Indigenous peoples occurs to achieve acceptance and adapt to a western culture [39]. However, some consider that university wellness programs and campaigns demonize drinking, without taking into account the cultural or social reasons why the Indigenous person does it [39].

Catholic and evangelical religious authorities have influenced drinking alcohol differently. The Catholic religion favored the prohibition of chicha [35] and maintained idealized and static historical imaginaries of the rural Indigenous [30]. Ritual celebrations, such as baptism and marriage have been a pretext for excessive alcohol consumption [35]. The evangelical religion brought with the missionaries new cultural practices and began a persecution of the Indigenous people who converted to the evangelical faith. The discontent of some members of the community due to the change in their ancestral practices led some Indigenous evangelicals to be forced to drink alcohol [42]. Also, conversion to this religion has led family members to stop drinking alcohol [42].

In Indigenous spirituality and traditional medical authority, substances are mixed to achieve states of trance and joy. In the celebration of rituals, they express love for nature and the strengthening of the spirit [35]. The ancestral doctor, before intervening a person with alcohol problems, may consume chicha, not as an alcoholic beverage, but to help open the mind [38]. They evaluate the person’s attitude and seek to convert negative energies into positive ones [32, 38]. Their spiritual powers have been questioned by the evangelical religion [42], but, they are interested in strengthening their position and benefiting from their ancestral knowledge in different areas [32, 34, 40].

#### Between transculturation and interculturality (TI)

Transculturation was reflected in the crisis and dynamism of culture (i.e., alive and in constant movement). The cultural crisis has been seen from the positive and negative effects generated on Colombian Indigenous peoples. The positive side of globalization was related to some young people who have seen improvements in their living conditions, a new social role, the diversification of their crops, and an interest in studying [35]. On the other hand, some displaced women say they feel better, in the sense of not being beaten by their drunken husbands and having a new role in their lives in the city [33].

On the negative side, there is the loss of values, a situation that has gone from solidarity to individuality [31, 35, 37], loss of authority and respect for the family, and the Indigenous councils [35, 36], and loss of the sense of chicha in the ritual, dietary and work aspects [35]. Cultural uprooting, loss of identity, and sense of belonging [31–37, 42].

The cultural crisis has generated both openness and resistance among Indigenous peoples. Openness is understood as enrichment with other Indigenous peoples [36] other cultures [35], religious practices [42], and the Indigenous of the urban area share their traditions without exclusions [30]. However, there is a struggle and resistance in defense of their own culture, as an effect of colonization [35]. There is a demand and transmission of ancestral values [30–36, 40]. The preservation of cultural practices, such as the preparation of chicha at home for drinking or sale among family and friends, without distinction of age and gender [35]. The preservation of daily memory [30, 32, 41] is reflected in expressions like this: “(…) events that happened in the daily life of the community are the ferment for what is known today as ‘the great chicha’, prepared by the people of the Muisca Indigenous council of Bosa” (researcher observation) [30].

The dynamism reflects how alive the culture is and the changes it has generated in the life of Indigenous peoples [35]. Among the changes in the vision of the world that young people have, who want to dress, speak, think, and behave like western culture [32, 35] due to the influence of the media, distant jobs [35], or roles as university students [39]. Children and young people follow stereotypes and adopt other behaviors in the city [33]. Drinking alcohol remains high in rural areas [35, 36] but increases in urban areas in marginal conditions [33]. This drinking, as well as the onset of sexual activity, occurs at an early age [36].

Likewise, the new generation of women does not accept repeating the mistreatment that their mothers have received from their drunken partners [42]. Also, the family constitution has changed due to the mixing of cultures and diverse joint celebrations [35]. In these, prejudices have been broken down, as Indigenous and non-Indigenous people begin to share spaces such as having a beer in a bar and listening to music [30]. Finally, there have been changes in individual and group values according to the interests of each culture [34], as well as the combination of ancestral and western medicine [35, 36].

Meanwhile, interculturality was lived in different ways. There is a lack of mutual agreements between community members and foreigners based on respect and recognition of each other’s traditions [37]. This can lead to complex situations such as the consumption of psychoactive substances [37]. Some participants expressed it as follows: “Now there are some young people involved in drug addiction, prostitution, drinking, etc. Let’s call it vices…” “… Well, the youth are opting for the life of foreigners. So, I think that this makes the natives go to nightclubs and bars, so all the young people lose their culture” [37]. Thus, it is seen as a phenomenon that puts at stake cultural and social values [35]. Also, it came to be seen as a destructive process for youth due to the influence it has had on drinking alcohol [36].

#### Paradoxes of the normalization of Alcohol

We start from the premise that society has certain reasons that give alcohol consumption the status of “normalization”, as well as the assignment of certain functions based on its needs. This normalization is explained by social acceptance [35, 39], the constitution as an ancestral practice [30, 33, 35, 42], and the legalization of consumption [35]. The functions of this consumption are diverse: sacred, labor, integration, economic, cultural, and food functions.

The sacred functions are related to ancestral rituals [32, 34, 35, 41], religious acts [32, 35], treatment for negative energies [38], and accompaniment at wakes and funerals [30, 34]. In labor functions, chicha is used to encourage work [32, 35], and increase physical capacity [34]. In the integration functions, alcohol is used in meetings and parties [30, 33, 35–37, 39]. For its part, in economic functions, alcohol is related to making profits from the sale of industrialized [35] or traditional [32, 35] beverages, which can be sold through informal street vending [32]. The revitalization of culture through orality [40] and cultural events was also evidenced [30, 32]. Likewise, they nourish themselves and calm their thirst with chicha [32, 35, 40, 41].

Types and patterns, predispositions, disharmonies, and harmonies in terms of alcohol consumption were presented. Three types of alcohol consumption were found: abusive drinking, low drinking, and non-drinking [30–42]. These three types share possible predispositions, and each has specific predispositions. The shared predispositions were: (a) the tradition of chicha and/or beer drinking in daily life [30, 33, 35] (b) parents and/or leaders drinking alcohol [32, 33, 35, 42] (c) drinking alcohol at home [32, 35] or in public [30–34, 42] and within intercultural [30, 33, 37] settings (d) being male or female [31–35, 38, 40, 42], (e) being of any age [30–42] and, finally, (f) individual willingness [35].

In the abusive type of drinking, the specific predispositions were related to considering excessive drinking as part of the culture [32, 33, 35]. This type of drinking is considered normal in the following expressions: “We say that chicha is culture, but if we overindulge it is not culture” (Indigenous leader) [32], “Cultural matters have led us to think that it is normal to consume alcoholic beverages, its use and abuse (…)” (community member) [35]. Also, the beginning of drinking at an early age [35, 36], the tensions caused by displacement [33], and that drinking is not conditioned to having the economic resources to purchase it [31, 32, 35].

Three subjective drinking patterns (regarding amounts, concentration, and frequency) that predispose to alcohol abuse were presented. Regarding amounts, they were described as indiscriminate or excessive [32, 34, 35, 42]. The alcoholic concentrations of the beverage were related to fermentation days [35] and being more intoxicating than rum and aguardiente (anise-flavored liqueur derived from sugar cane) [32] and, finally, the frequencies of consumption were expressed as “frequent” or “habitual” [32, 33, 35].

The disharmonies surrounding alcohol abuse were understood as negative. Examples of this negative view were: the presence of intra and intercultural conflicts [32, 34]; catastrophes; damage to themselves [32], destruction of their health [36], and violence towards others [32]; the existence of family and community problems [32]; gender and generational problems [31]; destabilization and deterioration of harmony and coexistence [32, 35]; lack of conditions to maintain unity, organizational process, resistance [32] and, finally, their existence as Indigenous peoples are put at stake [31].

Three types of disharmonies were found: socioeconomic, violence-related, and specific to children and youth. Socioeconomic disharmonies include problematic behaviors [32, 34, 35, 38]; community disorder and backwardness [32]; socio-ideological and economic control mechanisms [30, 32, 35]; loss of income and precariousness [42]; forgetfulness by the drinker of family and work responsibilities [32]; maintaining extramarital relationships; betting and gambling [42]; theft [32]; and, finally, poor community support if it is due to drunkenness [41].

Among the disharmonies related to physical and verbal violence, there was evidence of intrafamily violence [31, 33, 37, 40, 42]; sons who mistreat their parents [31, 32]; violence from women towards their partners [32, 35]; vulnerability in women due to violence exercised by men (including fathers, husbands, and brothers) [32, 35, 42]; increase in alcohol abuse and intrafamily violence in conditions of displacement given their tensions[33]; being single mothers and becoming pregnant at an early age[35]; abandonment and becoming widows [32]; feelings of sadness [31, 32, 34, 42]; separation from the partner due to mistreatment [33]; fear of their threats when reporting them to the authorities [31]; and, also, the generation of economic dependence of women on their partners, which generates another form of violence [42].

The studies found cases of alcoholism [32, 33, 35, 37, 38, 40, 42]; people who drink for no reason [35]; concomitant use of other drugs [31–33, 36–38, 42]; consumption related to prostitution [31, 37]; disability due to accidents [32] deaths due to suicide [32, 33, 35, 41] and, accidents on roads and bridges [32].

This is also evidenced by the number of complaints related to the problems of alcohol that the Indigenous authorities attend [31, 32]: “in the year they attend more demands for drunkenness problems than for other situations, exceeding a level of 120 cases” (Indigenous authority) [32]. The same occurs with other Indigenous peoples, in which the authorities “waste” time in these complaints [32].

Finally, disharmonies in children and young people were related to the mother who got drunk with the child breastfeeding [32]; children of drinking parents with malnutrition problems and low self-esteem; lack of attention; poor academic performance, and loss of the native language (which is exacerbated when the childhood authorities decide to remove the child from his or her family context) [32]; lack of affection [32, 40]; observing situations of mistreatment at home and in the community [32]; the father seen as an enemy and exclusive trust in the mother [31]; children and adolescents getting drunk [35] having sexual relations [36]; becoming pregnant [35], and sexual abuse [40]. Similarly, in contexts of displacement, or living in marginal areas of large cities, it is related to alcohol or drug abuse in places with the presence of violence and prostitution [33].

In the low-drinking type, the specific predispositions were community efforts to reduce excessive alcohol intake [32]. Concerning drinking patterns, it was found that: (a) the amounts were expressed as a little bit or “pocilladito” (small cup) [32, 35]; (b) the alcoholic concentrations were related to “drinking sweet water from sugar cane” [32]; and (c) the frequencies were described as drinking little [32, 35]. Harmonies were related to being calm, knowing how to dominate oneself [32, 35], and not having conflicts [32] or problems with anyone [35].

In the non-drinking of alcohol type, specific predispositions were related to the conversion to the evangelical religion of male drinkers and their families [42]. Also, they were related to the individual conscience of having trouble thinking and writing, and having better social opportunities [32, 42]. However, when the predisposition was given by individual conscience, there was a return to low drinking type [32].

The harmonies in the family, due to the evangelical conversion, were related to not wasting economic resources, with more educational and work opportunities, which favored upward social mobility [42]. Regarding women, the harmonies were related to finding refuge and hope; not suffering physical violence or being abandoned by a partner for extramarital relations; spending more time with the family, as well as being able to tell a different story to the women in their community [42]. Meanwhile, the harmonies generated by the individual conscience of the harm and having social opportunities were reflected in the recovery of their cognitive ability to write books and obtain a degree and specialization[32].

### Meta-theme. Alcohol: a chameleon that unpredictable society colors

Figure 3 represents the reinterpretation of the findings of primary studies through a visual display. It integrates the social processes previously described, through the symbolic use of the elements of Mother Nature, which together represent the meta-theme called “Alcohol: a chameleon that unpredictable society colors”. The chameleon (alcohol) receives from the environment (unpredictable society) indications that change the intensity and tone of its colors (responses), which are full of mystery and charm, like the human relationships in which drinking occurs.

The process called Dynamic Systems Mergers (DSM) (Indigenous and non-Indigenous system) when crossing and merging (*sun*) makes the interaction take different directions (*spider web*). The temperatures or energies generated in this encounter lead to tensions that can become heated, conflicting, and/or contradictory (*cloud with lightning*), but also become comforting (*relationship of the hummingbird with the flower*). This process, at the same time, permeates the social processes of Diverse Authority Spheres (DAS), Between Transculturation and Interculturality (BTI) and Paradoxes of the Normalization of Alcohol (PNA), in which these same tensions occur (*hummingbird-flower relationship or cloud with lightning*), and whose result, leads to a unique interaction with alcohol (*chameleon*). These social processes are the embodiment of dynamic systems (DSM) with distinct essences. In this constant interaction, there is a cyclic movement, in a floating environment itself (*water*).

The chameleon (*alcohol*) is a means that surprises with the magic of its colors, given by an unpredictable society, which determines the tonalities and intensities that can either harmonize, or disharmonize, Indigenous lives, within Colombian historical, social, cultural, economic, and political context.

### The epidemiological and socio-cultural profile of alcohol consumption among Indigenous peoples in Colombia

This profile presents the points of convergence and complementarity between the categories defined for the theoretical referents, which contain information on the social processes of the meta-synthesis to broaden the explanation of the phenomenon (Table 3).

**Table 3:** Epidemiological and socio-cultural profile of alcohol consumption among Indigenous peoples in Colombia (Source: Adapted by the authors, based on the proposal by Breilh and Men?ndez & Cortes)

SOCIOCULTURAL PROCESSES (Menéndez and Cortés)				CRITICAL PROCESSES (Breilh)		
**L**	Harmonies (balance) (+)		Disharmonies (unbalance) (-)		Harmonies (balance) (+)	**L**
**AP**	Cultural processes		Social consequences	Destructive processes	Protective processes	G**D**
	**DSM**: cosmogony and cosmovision; own education and medicine. **DAS**: religious and spiritual influence **BTI**: globalization: transculturation and interculturality.		**DSM**: poverty; food insecurity; armed conflict; globalization (westernization); weaknesses in health and education; lack of qualifications and job opportunities; extractivism; history of chicha (fermented drink) prohibition; homogenizing political management; socio-ideological and economic control. **DAS**: religious and educational influence **BTI**: colonization; globalization: transculturation, interculturality		**DSM**: historical self-sufficiency; their government; legalization of Indigenous reservations. **BTI**: globalization (educational and employment opportunities).	
**SG**	Cultural practices	Multifunctionality	Social consequences	Destructive processes	Protective processes	**PD**
	**DSM**: prevention with ancestral knowledge and care; application of traditional norms due to excessive drinking; stop drinking alcohol due to evangelical conversion; consumption in ritual celebrations; convert negative energies into positive ones. **BTI**: enrichment with other rural and urban cultures; preparation of chicha at home; demand and transmission of ancestral values; preservation of daily memory; a new generation of women do not accept mistreatment; cultural mixtures and celebrations; a combination of traditional and western medicine. **DAS/ PNA**: harmonies by evangelical conversion; no waste of money; women do not suffer physical violence; no infidelity, more family time; women tell a different story.	**PNA**: sacred, occupational, integration, profit-seeking, revitalizing culture, and food functions in ethnic groups.	**DSM**: precarious rural and urban housing; poor nutrition; forced displacement; loss of territories and cultures; school dropout; predominance of working-class in Indigenous ethnic groups in rural and urban; limited healthy activities sponsored by the alcohol industry; consumerism in general and drinking of industrialized alcoholic beverages. **DAS**: disruptive state norms in education and child-rearing, loss of authority; loss of the way in family roles; parents’ fear; leaders who facilitate alcohol drinking in the community and do not set an example; little attention, discussion, and follow-up; a contradiction in compliance with gender and generation equity norms; authoritarian or permissive education; preventive approach without the Indigenous context in universities; increased drinking in Catholic rituals; history of persecution of Indigenous evangelicals; questioned ancestral powers. **BTI**: from solidarity to individualism; loss of a sense of chicha in rituals, food, and work; cultural uprooting; loss of identity and sense of belonging; lack of agreements between cultures that influence drinking. High drinking in rural areas with an increase in urban areas in marginal conditions. **PNA**: alcohol abuse due to considering it part of the culture; beginning drinking at an early age; displacement tensions; and not being conditioned to having economic resources for its acquisition. Loss of income and forgetfulness of family and work responsibilities; infidelity; gambling; theft; poor community support if it is due to drunkenness; predominance of reports of drunkenness. Intra-family violence, among women, men, and children, increases with displacement. Mistreatment of women as something natural; single mothers and widows; separation from partners and threats for reporting them to the authorities; economic dependence on their partners as another form of violence. Multiple disharmonies in children of parents with abusive drinking. Prostitution, sexual abuse. Disability due to accidents, deaths by suicide, and accidents on roads and bridges.		**BTI**: displaced women not beaten by alcoholic partners and openness to new roles; work and study for young people (globalization). **PNA**: community efforts to reduce alcohol excess; no community problems; higher education opportunities.	
**I**			Individual consequences	Destructive processes	Protective processes	**ID**
			**PNA**: type of abusive drinking and their subjective drinking patterns; alcoholism; people who drink for no reason; use of other drugs; drunken breastfeeding; women with feelings of sadness and fear; loss of identity, beginning sexual relations, and pregnancy at an early age, following Western stereotypes.		**PNA**: type of low drinking and non-drinking and their subjective drinking patterns; being calm; self-control, the consciousness of the damage generated; recovering cognitive skills, writing books, obtaining a degree, and specialization.	

(Abbreviations: **L**: Level **AP**: Alcoholization Process **GD**: General society Dimension **SG**: Social Groups **PD**: Particular (groups) Dimension **I**: Individuals **ID**: Individual Dimension **DSM**: Dynamic Systems Mergers **DAS**: Diverse Authority Spheres **BTI**: Between Transculturation and Interculturality **PNA**: Paradoxes of the Normalization of Alcohol)

On the left side (Menéndez and Cortes) are the sociocultural processes of alcohol consumption. At the structural level, the alcoholization process (AP) reflects historically determined situations, in which power relations operate, either harmonizing (cultural processes) or disharmonizing (social consequences) the the Indigenous people’s life. This dynamic then becomes evident at the level of social groups (SG). Either with influences that have favored harmony in their ways of life, in which they have expressed their cultural practices and multiple positive functions that they give to alcohol, or on the contrary, have generated indirect negative social consequences (disharmonies). These, as well, have influenced the level of the individuals (I) with the presence of negative consequences, which have directly affected the health of individuals.

On the right side (Breilh), the critical processes of alcohol drinking are articulated. At the structural level, the general society dimension (GD) reflects the protective (harmonies) and destructive (disharmonies) processes that have structured the lives of society. These influences have been reproduced at the level of the ways of life of the Indigenous groups (PD), who are the ones who have lived through these processes, and, at the same time, struggle to preserve their identity. Social class, ethnicity, gender, and age concerning drinking alcohol determined the differences between the processes. At the same time, in the individual dimension (ID), the final impacts determined by society have been manifested.

## Discussion

This meta-synthesis analyzed qualitative research findings on alcohol consumption among Indigenous peoples in Colombia from 2004 to 2019. The reinterpretation of the findings identified the social processes that influenced the interaction with alcohol: Dynamic Systems Mergers, Diverse Authority Spheres, Between Transculturation and Interculturality, and the Paradoxes of the Normalization of Alcohol (Fig. 2). Each social process was analyzed with evidence from the literature.

Likewise, an Epidemiological and socio-cultural profile of alcohol consumption among Indigenous peoples in Colombia was obtained. This resulted from the integration of the social processes (identified in meta-synthesis) with the predefined categories for the social determination of health and sociocultural epidemiology perspectives. The analysis of whether this is an adequate way to explain alcohol consumption among Indigenous peoples in Colombia is also explained.

### Dynamic Systems Mergers

Tensions between the Indigenous and non-indigenous systems have been historical. The history of the prohibition of chicha in Colombia began in the first half of the 20th century with an anti-alcohol campaign [53] This was considered a racist and discriminatory struggle whose stigma fell on vulnerable groups such as Indigenous people belonging to the lower social classes [53]. These conditions persist in rural and urban areas in the ethnic groups of this study.

Likewise, the never-ending history of armed conflict and mining exploitation have been linked to alcohol consumption. There is evidence in people in a situation of forced displacement, caused by conflict, of an increase in alcohol consumption as a way of channeling anguish and existential problems [54], which coincides with the findings of this meta-synthesis. On the other hand, there is evidence in the literature of the relationship between mining work and alcohol consumption [55]. This is evidenced in the offer of alcoholic beverages to Indigenous people by the owners of the mines, to later deduct it from their wages [55], which makes alcohol an instrument in the generation of debt and the maintenance of forced labor relations [20]. This human exploitation dates to colonial times when Spaniards sponsored alcohol as a weapon to calm Indigenous discontent [56]. However, in the articles included in this meta-synthesis, this relationship was superficially addressed in only one study [32].

While some people can make healthy choices about drinking alcohol, their choices [57] and the magnitude of harm [7] are framed by the social conditions in which they live [57]. In Indigenous peoples living in precarious conditions, there is the presence of health problems and early deaths [20]. This disadvantage along with abusive drinking of alcohol influences the presence of greater harm to Indigenous peoples [58], which was evidenced in the multiple disharmonies identified.

### Diverse Authority Spheres

Family, school, church, and government spheres influence the preservation of diverse aspects that characterize cultures [59]. This dynamic is corroborated by the findings of this meta-synthesis, in which there were powers of greater or lesser degree that influenced the drinking, or not, of alcohol, as well as the presence of precepts to validate or condemn behavior [59]. These were differences between Indigenous and non-Indigenous authorities, which have led to conflicting and/or contradictory relationships around alcohol consumption in different groups and individuals in society [55, 60–65].

### Between Transculturation and Interculturality

Transculturation comes from a colonial historical context between a dominated and a dominant culture, which has conditioned the cultural dynamics around alcohol [66] and continues to do so with globalization within the framework of capitalism. This has generated losses and cultural uprooting in Indigenous communities and an increase in alcohol abuse [66–68] as a response to the history of domination and the current social conditions experienced by various Indigenous peoples around the world [57, 60, 66, 69–72].

Nevertheless, this process has led to moments of reciprocal exchange with other cultures [66], which was also present in some of the findings of this meta-synthesis. This confirms the cultural hybridity, in which social, cultural, and ethnic elements [73] have been mixed, generating changes, gains, and, at the same time, struggles to reclaim their own. As well as the continued existence of ancestral Indigenous practices such as the preparation of traditional fermented beverages [72, 74, 75]. All this is experienced by some Indigenous peoples in rural and urban areas.

Meanwhile, interculturality seeks the possibility of dialogue between cultures and the construction of different societies [76]. However, in some of the findings of this meta-synthesis, it was perceived as facilitating the loss of Indigenous customs and the absence of mutual agreements, which shows divergences between the expected ideal and reality.

### Paradoxes of the Normalization of Alcohol

Alcohol drinking is normalized because it is considered part of everyday life in many societies [20, 56]. In Latin American and world communities, alcohol fulfills positive functions [20, 21, 56] and appears as “necessary” [20, 77] which coincides with the Indigenous groups in this study.

The type of alcohol abuse was the most repetitive. Subjective patterns of this type of drinking (regarding amounts, frequency, and concentration) have also been presented in quantitative studies with Indigenous cultures. A study in a South American Indigenous group found higher trends in all patterns in men [1]. Higher prevalence of problem drinking (86.5%), frequency (92.3% reporting consumption of six or more drinks usually monthly), and consequences from their drinking (80.1% classified as harmful drinkers) [1].

The same study revealed that industrialized beverages have gradually replaced traditional beverages. Industrialized beverage frequency intervals have been higher, as well as negative social consequences [1]. The findings of this study present a similarity between the patterns of drinking mentioned and those observed among the Native American population in the United States [1]. In addition, another study in Latin America evidenced a high moderate alcohol consumption among Indigenous men in urban areas [74], which is consistent with some of the cases in this study.

Shared predispositions were present in the types of abusive, low, and non-drinking alcohol. The same situation, or a combination of several situations, led people to any of the three types of drinking. This is related to the positive or negative learned predispositions that are manifested in people’s attitudes and beliefs towards alcohol consumption [77] and that are susceptible to being modified by the cultural dynamism in which the Indigenous ethnic groups live.

The alcohol abuse presented predispositions corroborated in the literature. Considering it as normal means that it is not seen as a problem [20, 56, 78]. Exposure to alcohol consumption at an early age increases four times the probability of presenting abusive consumption in adulthood [79] a situation that occurs in Indigenous people in Latin America [63, 69] Australia, Canada, and the United States [70, 78]. This, in turn, increases even more in conditions of displacement (the situation of some Indigenous living in urban areas) given the stress.

[67] and anguish [54] that it generates.

Also, having, or not, having economic resources [7, 22] does not impede alcohol abuse. This predisposition, in part, is presented in a context in which traditional beverages are prepared at home (fermentation of foods such as corn, and sugar cane, among others), with limited resources and with the potential to generate alcohol abuse, which was evidenced. However, this situation has led to racist scientific interpretations throughout Colombian history [80]. These interpretations tried to demonstrate a link between the fermentation of traditional beverages (chicha), with the emergence of a specific pathology, attributed to Indigenous and half-breeds (an offensive way of describing any person of mixed race) [80].

It is essential not to fall back into stigmatization related to alcohol abuse among Indigenous peoples. Traditional beverages, as well as industrialized ones, have been an instrument present in the relationships and tensions of the social processes identified. In this study, this social dynamic was reflected more in disharmonies, in which the excess of traditional (high fermentation) and industrialized beverages was present in the lives of Colombian Indigenous peoples.

Many policies have attempted to control alcohol consumption in the general population. In the case of industrialized beverages, there is a broad evidence base demonstrating the effectiveness of minimum unit pricing to reduce alcohol consumption and alcohol-related harm [81]. However, the findings of this study would make in part (traditional beverages fermented at home) this policy ineffective for Indigenous peoples. This requires alternative policies with better socio-cultural, economic, and territorial pertinence, and in which history is known and does not repeat past mistakes [80].

The multiple disharmonies presented have been defined as social, spiritual, mental, and physical unbalances [68]. These occurred in environments with the presence of poverty [8] and limited opportunities. This corroborates the existence of a greater disadvantage for the Indigenous ethnic groups in this study, in terms of the magnitude of the damage and death [7, 20, 60]. The positive functions granted to alcohol consumption became contradictory and conflicting [21] insofar as they generated negative social consequences [20]. Various studies, with other Indigenous peoples, confirm the findings related to socioeconomic disharmonies [20, 60, 64], related to violence [60, 62, 63, 65], and those specific to childhood and youth [55, 60, 61, 63, 67, 82] which may affect the continued existence of the Indigenous.

Harmonies have been defined as favoring balance and the presence of daily behaviors based on care and respect [68]. There are Indigenous communities that have avoided alcohol abuse by converting to the evangelical religion in favor of community well-being [63, 83]. While in others there seems to be an individual [84] and/or community [58] non-religious interest in reducing drinking. Furthermore, the positive influence of having real social opportunities, as well as the power to choose to enjoy a healthy, full, and lasting life [85, 86], is consistent with a case presented in a study of this meta-synthesis [32].

### Meta-synthesis. Alcohol: a chameleon that unpredictable society colors/ The Epidemiological and socio-cultural profile of alcohol consumption among Indigenous peoples in Colombia

The results of this meta-synthesis support the social determination of health perspective [14] and its complementarity with sociocultural epidemiology [15]. This is an adequate way of explaining the social processes that influence interaction with alcohol among Indigenous peoples in Colombia (Table 3). In the dimensions of this epidemiological and socio-cultural profile, contradictory social processes and power relations were identified. This was observed from the structural dimension that permeates the ways of life and lifestyle of the Indigenous peoples (who have relative autonomy) [18, 87].

This profile shows how harmonies and disharmonies occurrence are influenced by power relations. It was possible to identify power relations such as class, gender, ethnicity [17, 18], and age. Ultimately, they make it possible to identify a differential epidemiological distribution [17, 18] around drinking or not drinking alcohol in Indigenous peoples. (Table 3)

Future research on alcohol consumption among Indigenous peoples living in rural and urban areas should deepen on aspects such as the dynamics of transculturation and interculturality, the influences of specific authorities, types, and patterns of drinking, and predispositions. Research on the disharmonies and harmonies encountered, in which women, children, and young people can be prioritized, should be carried out. It is recommended to make use of participatory action research, as well as quantitative research, and perspectives such as the social determination of health and sociocultural epidemiology. Finally, to advance in the generation of evidence-based practice that informs public health interventions with Indigenous peoples that can then be evaluated.

### Strengths and limitations

Alcohol consumption among Indigenous people has been discussed in Latin American qualitative research for decades [69, 71, 88, 89]. The main strength of this research is that, to our knowledge, there are no previous meta-synthesis studies that sought to integrate and reinterpret the findings of this phenomenon in Indigenous Colombian ethnic groups. Therefore, it provides relevant evidence to reflect, guide, and act on this situation.

However, the study had several limitations. First, the studies reviewed corresponded to a specific time range (2004–2019) and therefore do not include more past and recent literature. Second, although the research was retrieved from different electronic sources, some may have been missing, adding that print sources were not included, which may have resulted in the lack of other ethnic group literature. Even with these limitations, it was possible to capture the phenomenon broadly, expand knowledge and open the field for new research.

## Conclusion

This meta-synthesis makes the understanding the alcohol consumption among Indigenous peoples in Colombia broader and deeper. There have been contradictory processes related to drinking, or not, alcoholic beverages. This study found that alcohol consumption is a social construction. Alcohol acts as an instrument present in the changing relationships and tensions given in social processes. The social processes identified (Dynamic Systems Mergers; Diverse Authority Spheres; Between Transculturation and Interculturality; and Paradoxes of the Normalization of Alcohol) are reflected in harmonies, or disharmonies, in the lives of Indigenous Colombians who are part of a historical, sociocultural, economic, and political context.

The articulation of its results to a social determination of health and sociocultural epidemiology framework provided an adequate explanation of alcohol consumption in Indigenous peoples in Colombia. These perspectives made it possible to rethink social processes. Allowed the recovery of the complexity of the health-disease process, and the influence of power relations, which result in a differential epidemiological distribution. Capturing relevant aspects of reality such as these contribute to more contextualized public health, practice, policies, and programs.

It is necessary to read the reality of alcohol consumption among Indigenous peoples from the dimensions of people’s life that influence in contradictory ways the health and disease processes of groups and individuals. The generation of alternative or complementary policies on alcohol consumption among the Indigenous peoples in Colombia needs to be open to diverse knowledge perspectives. These policies should provide different possibilities for a phenomenon that does not have unique answers.

## Electronic supplementary material

Below is the link to the electronic supplementary material.

Additional file 1: Literature Search Strategy

Additional file 2: Critical Appraisal Form Guidelines: qualitative studies, based mainly on Lincoln Y, Guba E [45]

Additional file 3: Findings Classification. Example of typology of findings in qualitative studies, based on Sandelowski M, Barroso J [24, 48]

## Data Availability

All data analyzed for this study are included or referenced in this published article [and its supplementary information files].

## Abbreviations

ENTREQ: Enhancing Transparency in Reporting the Synthesis of Qualitative research
DeCS: Descriptors in Health Sciences
EMTREE: Embase Subject Headings
MeSH: Medical Subject Headings
DSM: Dynamic Systems Mergers
DAS: Diverse Authority Spheres
BTI: Between Transculturation and Interculturality
PNA: Paradoxes of the Normalization of Alcohol
AP: Alcoholization Process
GD: General society Dimension
SG: Social Groups
PD: Particular(groups) Dimension
I: Individuals
ID: Individual Dimension
