# Supplementary material for: Social determination of alcohol consumption among Indigenous peoples in Colombia: a qualitative meta-synthesis
Source: BMC Public Health. 2023 Mar 13;23:478. doi: 10.1186/s12889-023-15233-6 (PMC10009970; doi:10.1186/s12889-023-15233-6)
Supplement: Supplementary file 1 — Additional file 1: Literature Search Strategy [file 12889_2023_15233_MOESM1_ESM.docx]

**Additional file 1: Literature Search Strategy (September to October 2020)**

| **Database** | **Search terms** | **Search restrictions** | **Number of records** |
| --- | --- | --- | --- |
| SCOPUS | "indigen*" AND "colombia" | Only all fields, limit to: all open access, 2004 to 2019, colombia, articule and review, English and Spanish, Journal: Revista Colombiana de Antropologia, Revista de Estudios Sociales, Antipoda, Revista de Salud Publica, Colombia Internacional, Salud Uninorte, Colombia Medica, Cuadernos de Desarrollo Rural, Revista Facultad de Medicina, Revista Panamericana de Salud Publica Pan American Journal of Public Health, Universitas Psychologica,Nomadas,Revista Ciencias de la Salud, Cultura de los Cuidados, International Journal of Environmental Research And Public Health, Investigacion y Educacion en Enfermeria, Revista Colombiana de Sociologia, Revista de Antropologia Social,Aquichan, Avances En Psicologia Latinoamericana, BMC public health, Ciencia E Saude Coletiva, Enfermeria Global, European Review of Latin American and Caribbean Studies,Frontiers in Public Health,International Journal for Equity in Health | 164 |
| PROQUEST | ("indigenous people$" OR "indigenous" OR "Aboriginal$" OR "native$" OR "ethnic$") AND ("Alcohol drinking" OR "alcoholic beverages" OR "Alcohol use" OR "alcohol$" OR "chicha" OR "liquor") AND "colombia" | Advanced search, Only full text, limit to 2004 to 2019, cualquier campo, tipo de fuente: revistas cientificas, revistas de caracter general, revistas profesionales,  tesis doctorales y tesinas. Tipo de documento: articulo, articulo principal, disertaciones/ tesis. English and Spanish. Limit to Colombia.Excluir documentos duplicados. | 145 |
| LILACS | indigen* AND colombia | Only full text. 2004 to 2019. English and Spanish. By title, abstract, subject | 372 |
| JSTOR | indigen* AND (alcohol OR chicha) AND colombia | Only all fields, for content I can access, articles  Only 2004 a 2019,  Only subjects: Anthropology, cultural studies, gender studies, psychology, public health, religion, public health, sociology | 90 |
| **Web search engine** | **Search terms** | **Search restrictions** | **Number of records** |
| Google Scholar  (first 10 sheets) | ("indigenous people*" OR "indigenous*" OR "Aboriginal" OR "native" OR "ethnic group*" OR "ethnic") AND ("Alcohol drinking" OR "Alcohol consumption" OR "alcoholic beverages" OR "Alcohol use" OR "alcohol intake" OR "liquor") AND "colombia" | Only 2004-2019. English and Spanish, by relevant, exclude citations, include patents | 200 |
|  | ("indígena" OR "indigena*" OR "étnico*" OR "pueblo* indígena*" OR "aborigen*") AND ("chicha" OR "alcohol" OR "consumo de alcohol" OR "trago" OR "licor") AND "colombia" | Only 2004-2019. English and Spanish, by relevant, exclude citations, include patents | 420 |
|  | ("indígena" OR "indigena*" OR "étnico*" OR "pueblo* indígena*" OR "aborigen*") AND ("chicha" OR "alcohol" OR "consumo de alcohol" OR "trago" OR "licor") AND "colombia" AND ("determinación social de la salud" OR "determinacion social de la salud") | Only 2004-2019. English and Spanish, by relevant, exclude citations, include patents | 78 |
|  | ("wayuu" OR "pijao" OR "pastos" OR "Zenú" OR "sikuani" OR "inga" OR "cubeo") AND ("chicha" OR "alcohol*" OR "consumo de alcohol" OR "trago" OR "licor") AND "colombia" | Only 2004-2019. English and Spanish, by relevant, exclude citations, include patents | 320 |
|  | ("embera") AND ("chicha" OR "alcohol*" OR "consumo de alcohol" OR "trago" OR "licor") AND "colombia" | Only 2004-2019. English and Spanish, by relevant, exclude citations, include patents | 80 |
| **Academic Institutional Repositories** | **Search terms** | **Search restrictions** | **Number of records** |
| FLACSOAndes Repository | indigen* AND (alcohol OR chicha) AND colombia | Only Todo FLASCO  Only materias: colombia | 74 |
| CINDE Repository | indigen* AND colombia | Only all of Dspace | 20 |
| URACCAN Repository | indigen* AND (alcohol OR chicha) AND colombia | Only all of Dspace | 18 |
| Uniandes Repository | "indigen*" AND ("alcohol*" OR "chicha") AND "colombia" | Only 2004 to 2019 Only trabajos de grado-pregrado- maestria -doctorado | 39 |
| UNAL Repository | indigen* AND ("alcohol*" OR "chicha") AND "colombia" | Only all of Dspace | 55 |
| UDEA Repository | indigen* AND (alcohol* OR chicha) AND colombia | Only all of Dspace  Only 2004 to 2019 | 11 |
| Universidad del Cauca Repository | indigen* AND (alcohol* OR chicha) | Only all of Dspace | 1 |
| Universidad del Valle Repository | ("indigen*" OR "misak") AND ("alcohol*" OR "chicha") AND "colombia" | Only 2004 to 2019 | 28 |
| **Journals** | **Search terms** | **Search restrictions** | **Number of records** |
| Revista Colombiana de Psiquiatria. (EBSCOhost) | ("indígena" OR "indigena*" OR "étnico*" OR "pueblo* indígena*" OR "aborigen*") AND ("chicha" OR "alcohol" OR "consumo de alcohol" OR "trago" OR "licor") AND "colombia" | Only full text. English and Spanish. desde que los recopilan 2009 a 2019. Since they were collected from 2009 to 2019 | 15 |
| Revista de la Facultad de Medicina de la UNAL. (EBSCOhost) | indigen* | Only full text. English and Spanish. desde que los recopilan 2009 a 2019. Since they were collected from 2009 to 2019 | 35 |
| Revista Facultad Nacional de Salud Pública. Universidad de Antioquia. (website) | indigen* | Only Advance Research. Limit to 2004 -2019 | 27 |
| Revista de Estudios Sociales. Universidad de los Andes. (website) | ("indígena" OR "indigena*" OR "étnico*" OR "pueblo* indígena*" OR "aborigen*") AND ("chicha" OR "alcohol" OR "consumo de alcohol" OR "trago" OR "licor") AND "colombia" | Only Advance Research. Limit to 2004 -2019 | 22 |
| Revista Antídopa. Revista de Antropología y Arqueología. Universidad de los Andes. (website) | ("indígena" OR "indigena*" OR "étnico*" OR "pueblo* indígena*" OR "aborigen*") AND ("chicha" OR "alcohol" OR "consumo de alcohol" OR "trago" OR "licor") AND "colombia" | Only Advance Research. Limit to 2004 -2019 | 24 |
| Revista Ciencia e Interculturalidad. Universidad de las Regiones Autónomas de la Costa Caribe Nicaragüense (URACCAN) (website) | colombia | Only Advance Research. Since journal started from 2008 to 2019 | 30 |
