# Supplementary material for: Social determination of alcohol consumption among Indigenous peoples in Colombia: a qualitative meta-synthesis
Source: BMC Public Health. 2023 Mar 13;23:478. doi: 10.1186/s12889-023-15233-6 (PMC10009970; doi:10.1186/s12889-023-15233-6)
Supplement: Supplementary file 2 — Additional file 2: Critical Appraisal Form Guidelines: qualitative studies, based mainly on Lincoln Y, Guba E [45] [file 12889_2023_15233_MOESM2_ESM.docx]

**Additional file 2: Critical Appraisal Form Guidelines: qualitative studies, based mainly on Lincoln Y, Guba E. [45]**

| **Criteria** | **Strategies appraisal** |
| --- | --- |
| **Credibility:**  How do the findings represent the views of the participants? | Persistent observation |
|  | Prolonged engagement  in the field |
|  | Triangulation (methods, sources, researchers, theories) |
|  | Member checking |
|  | Structural coherence |
|  | Referential adequacy |
|  | Peer debriefing |
|  | Using quotes |
| **Transferability:**  How applicable are the findings to other groups of people and other context? | Thick description |
|  | Comparison of sample to demographic data |
| **Dependability:** Would the findings be repeated if the inquiry were replicated with the same (or similar) subjects in the same or similar context? | Overlap methods |
|  | Stepwise replication |
|  | Inquiry audit |
| **Confirmability:**  How can we be certain that the findings have been determined by the subjects and contexts of the inquiry, rather than researchers’ interests? | Audit trail of synthesis products |
|  | Fieldwork materials and evidence |
|  | Peer debriefing |
| **Complementary criteria** | **Aspects appraised** |
| **Ethics:**  How do researchers approach ethical aspects? | Informed consent (individual/community) |
|  | Risks and benefits |
|  | Knowledge of the purpose |
| **General Relevance:**  Does the research provide a useful contribution? | Useful contribution |

| **Strategies appraisal** | Define for each strategy: Presence (yes/no), Relevance (yes/no) and write reviewer's observations and comments (reasons of judgment) |
| --- | --- |
| **Overall appraisal** | |
| General comments on research appraisal | Description of the analysis performed (reasons of judgment) |
| Considering the aim of the research and the value of the findings,  Do you recommend including the research in the meta-synthesis? | yes/no |
| Do you question anything about the research? | yes/no, explain why |
| Consensus | yes/no |
| Date | day/month/year |

* The findings classification phase helps to achieve a balance in this appraisal process.
