# Supplementary material for: Social determination of alcohol consumption among Indigenous peoples in Colombia: a qualitative meta-synthesis
Source: BMC Public Health. 2023 Mar 13;23:478. doi: 10.1186/s12889-023-15233-6 (PMC10009970; doi:10.1186/s12889-023-15233-6)
Supplement: Supplementary file 3 — Additional file 3: Findings Classification. Example of typology of findings in qualitative studies, based on Sandelowski M, Barroso J [24, 48] [file 12889_2023_15233_MOESM3_ESM.docx]

**Additional file 3: Findings Classification. Example of typology of findings in qualitative studies, based on Sandelowski M, Barroso J [24,48].**

| **Extraction of raw data and findings** | | **Typology of findings** | | | | |
| --- | --- | --- | --- | --- | --- | --- |
| **Direct quotations from the participants (raw data)** | **Findings from primary authors** | **No finding** | **Topical survey** | **Thematic survey** | **Conceptual/thematic description** | **Interpretive explanations** |
| Cod. 1-6 "I'm going to the party, the motivation I have to attend this party is that I can have a few drinks with my friends. Besides, I had nothing else to do, so I decided to come and have some fun. Since they said there were first communions I came first to the mass, but now I am going to the party with my sister and other friends. There are quite a few of us and at Carlos' place, the party is good. There are a lot of people from all over; it's good for dancing and getting company" | Religious festivities are also part of the life of the community members, where they first attend religious services and then go to the houses that celebrate baptisms, first communions, or marriages. Generally, the parties are attended by many people from the community regardless of age. |  |  | x |  |  |
| Cod. 1-42"A distinction is made at the time of drinking because they are legally accepted and shared naturally in the community, while industrialized beverages have been brought to the community for economic purposes and are not welcomed to be distributed in the community's cultural events." | The beverages in the community have two special characteristics: the first one is related to traditional beverages, which are called in this way for having been elaborated by the families and preserved the knowledge from generation to generation; the second one refers to industrialized beverages such as Aguardiente Caucano, Ron Viejo de Caldas, beer and other beverages, brought to the different activities with a double purpose: one, the celebration of every party there is, and the other for the search of profit: One, the celebration of every party there is, and another for the search for profit; being then when the community authority intervenes and pretends that the commercialization of these products is not done, since it is equivalent to cultural changes, which has made that young people already prefer industrialized beverages. |  |  |  | x |  |
| Cod.10- 1 "Well, in the age of youth, what one seeks is identification, to identify with any group, regardless of what it is." | On the other hand, influence and friendships play a factor in the adherence and acceptance of indigenous students as a mechanism of adaptation to life in Western culture. All this is because young people seek to identify themselves in a place or society and in the different groups to which they belong, and consumption of psychoactive substances is presented as a means of achieving this acceptance. (...) These affirmations demonstrate that in the attempts to generate bonds and ties of acceptance for adaptation, the indigenous student restructures his behavior and modifies cultural aspects for recognition. |  |  |  |  | x |
